# Preparation and Application of Standardized Typical Volatile Components Fraction from Turmeric (*Curcuma longa* L.) by Supercritical Fluid Extraction and Step Molecular Distillation

**DOI:** 10.3390/molecules23071831

**Published:** 2018-07-23

**Authors:** Guang-Ping Lv, De-Jun Hu, Yan-Qing Zhou, Qing-Wen Zhang, Jing Zhao, Shao-Ping Li

**Affiliations:** 1 State Key Laboratory of Quality Research in Chinese Medicine, Institute of Chinese Medical Sciences, University of Macau, Macao 999078, China; guangpinglv2012@hotmail.com (G.-P.L.); hdjred@gmail.com (D.-J.H.); yb27511@umac.mo (Y.-Q.Z.); QWZhang@umac.mo (Q.-W.Z.); 2 School of Pharmaceutical Sciences, Nanjing Tech University, Nanjing 211800, China

**Keywords:** *Curcuma longa*, turmeric, quality control (QC), standardized typical volatile components fraction, molecular distillation

## Abstract

A green and reliable method using supercritical fluid extraction (SFE) and molecular distillation (MD) was optimized for the separation and purification of standardized typical volatile components fraction (STVCF) from turmeric to solve the shortage of reference compounds in quality control (QC) of volatile components. A high quality essential oil with 76.0% typical components of turmeric was extracted by SFE. A sequential distillation strategy was performed by MD. The total recovery and purity of prepared STVCF were 97.3% and 90.3%, respectively. Additionally, a strategy, i.e., STVCF-based qualification and quantitative evaluation of major bioactive analytes by multiple calibrated components, was proposed to easily and effectively control the quality of turmeric. Compared with the individual calibration curve method, the STVCF-based quantification method was demonstrated to be credible and was effectively adapted for solving the shortage of reference volatile compounds and improving the QC of typical volatile components in turmeric, especially its functional products.

## 1. Introduction

Since the Japanese scientific academic community defined the class of "functional foods"— conventional and modified foods that included additional health benefits beyond basic nutrition in the early 1980s, the foods with health benefits became more and more popular in the daily diets of individuals [1]. Turmeric, powder of *Curcuma longa* L. rhizome (Zingiberaceae), is widely used as a food additive (for its flavor and yellow color), dietary supplement, and medicine [2,3]. It is officially documented as a medicine and food dual purposes items by the National Health Commission of the People’s Republic of China [4] and as Dietary Supplement by the United States Pharmacopeia (USP) [5]. The main components in *C. longa* are curcuminoids and essential oil. Essential oil has been revealed to possess multiple pharmaceutical activities including hyperlipidaemia amelioration [6], antibacterial [7], antioxidant [8,9], anti-inflammatory [10], antidiabetic [11] and bone-protective effects [12]. However, even in the Chinese Pharmacopiea (ChP) and USP, there is no effective quality control (QC) method for essential oils in *C. longa* rhizomes. However, it was noted that there are significant variations both in constituents and contents of essential oils in *C. longa* rhizomes from different geographical locations [13]. Additionally, turmeric products including curry, turmeric anti hangover drink cans, functional turmeric powder, and turmeric tablets, etc. have been variously marketed globally. The extensive consumption also significantly stimulates adulteration. Therefore, the QC is of crucial importance for ensuring its safety and efficacy in biomedical and functional foods usage.

Reference compounds, the standards for qualitative and quantitative analysis of analytes, are crucial in chemical analysis [14,15]. In fact, purified reference compounds are usually difficult to obtain. Separation and purification of pure volatile components are especially challenging due to their structural similarity, strongly hydrophobic properties, and poor stability. Column chromatography over silica gel, which is broadly used in the separation of components from natural materials, is not very effective in the separation of volatile components. High-speed counter-current chromatography (HSCCC) [16], high-performance centrifugal partition chromatography (HPCPC) [17,18], and preparative GC (Prep-GC) [19] have been successfully used in the separation of the volatile components from *Curcuma* species. However, some pure chemical compounds are still difficult to obtain because of their instability and/or very low amounts. Furthermore, volatile components are particularly thermo- and photo-labile, which makes them difficult for storage after the purification. Based on previous studies ar-curcumene, the main volatile component in *C. longa*, could be degraded within 2 months even when stored at −20 °C. However, it is stable in the crude essential oils [18]. Therefore, alternative methods without reference compounds are beneficial for the QC of volatile components. 

**Figure 1:**
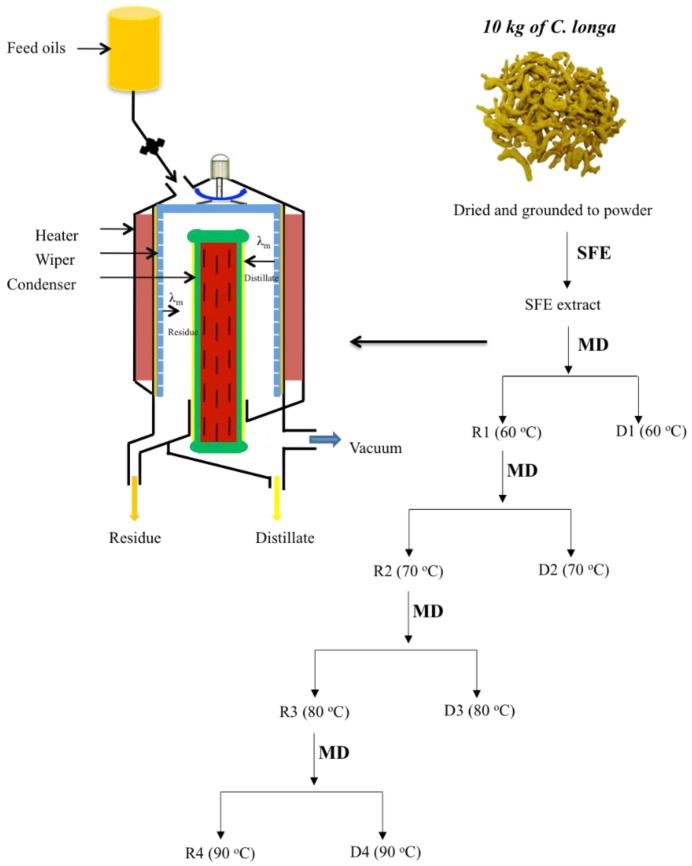
Schematic diagram of wiped film molecular evaporator and scheme of sequential distillation performed by molecular distillation. D1–4: Distillate 1–4, R1–4: Residue 1–4.

Molecular distillation (MD) or short path distillation is a comparatively new separation technology. It can separate liquid–liquid mixtures at temperatures far lower than the boiling point by the difference of mean free path (λ_m_) of molecules under high vacuum condition. It allows for concentrating particular compounds with no deterioration of their natural properties [20]. During the distillation, the distance between evaporating and condensing surfaces is less than the λ_m_ of the molecules involved [21]. Different substances can be separated from mixed components according to their λ_m_ (Figure 1). It is an appropriate method for the separation and purification of thermally unstable materials with low vapour pressures and without the danger of thermal decomposition [22]. 

In this study, a green and reliable method based on SFE and MD was developed for the preparation of STVCF from *C. longa*. Its application, i.e., STVCF-based qualification and quantitative evaluation of major bioactive analytes by multiple calibrated components, was proposed to easily and effectively control the quality of volatile components in *C. longa*. The feasibility and credibility of this methodology were further assessed with a developed fast GC-MS method.

## 2. Results and Discussion

### 2.1. Conditions for Supercritical Fluid Extraction (SFE) of Essential Oil

**Figure 2:**
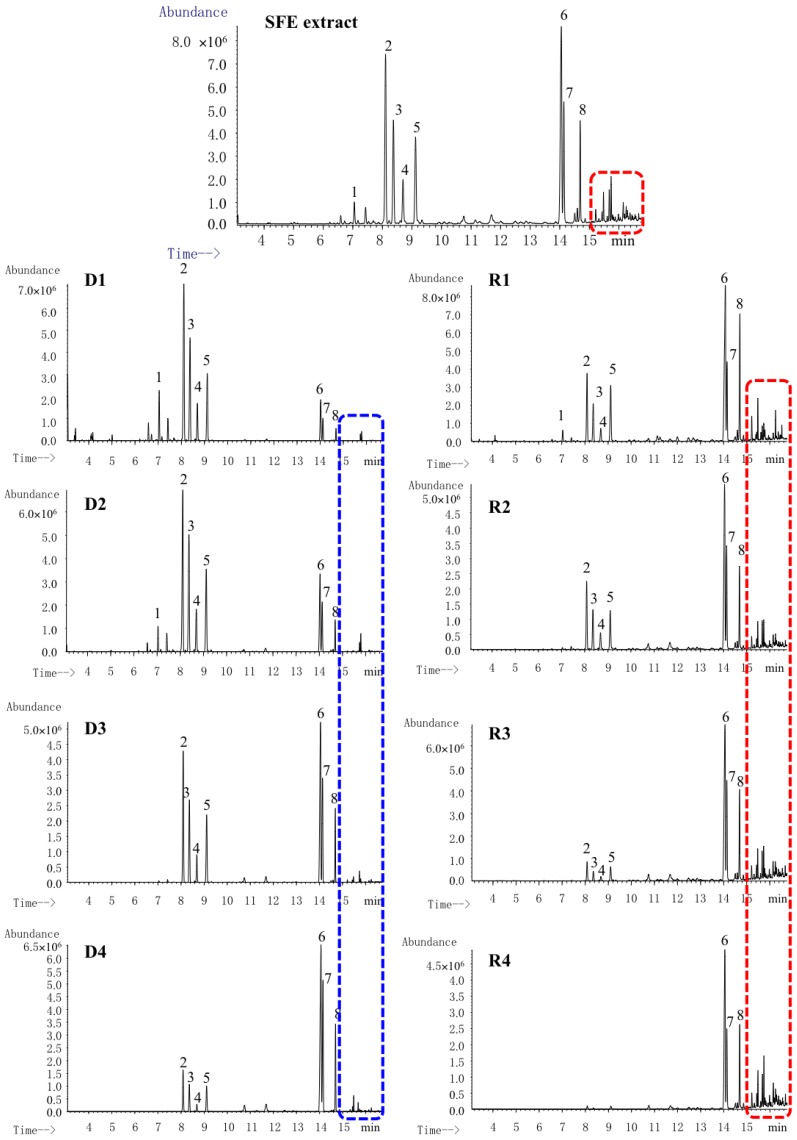
GC-MS total ion chromatograms of supercritical fluid extraction (SFE) extract and different distillates of molecular distillation. D1–4: Distillate 1–4, R1–4: Residue 1–4; 1. β-caryophyllene, 2. ar-curcumene, 3. zingiberene, 4. β-bisabolene, 5. β-sesquiphellandrene, 6. ar-turmerone, 7. α-turmerone, 8. β-turmerone.

SFE is regarded as a green process because it does not use organic solvents with adverse environmental impacts. The parameters, including extraction pressure, percentage of modifier, and extraction temperature, significantly influence the extraction efficacy of SFE, in which the extraction pressure and percentage of modifier play the most important roles [23,24]. Higher pressure is beneficial for increasing the yield, in which significant amounts of waxes or polar compounds are co-extracted, and consequently, the essential oil content in the extract decreases [25]. This phenomenon is quite common in the extraction of natural products. If high pressure and temperature (500 bar and 50 °C) are applied, the main extracted compounds from marigold are triterpenoid esters [26], while if lower pressures and temperature (200 bar and 40 °C) are applied, the produced extract is enriching in aliphatic hydrocarbons, acetyl eugenol, and guaiol [27]. On the other hand, modifiers are commonly added to CO_2_ to increase its solvent power toward polar molecules. However, the addition of a modifier to attain a suitable recovery of essential oil is not necessary. Several studies showed that extraction yield increased with the percentage of modifier increased, but this also causes a reduction of the terpene compounds content and an enhancement of polar components (such as phenolic acid) in the extracts [28,29]. Additionally, to suppress co-extraction of higher molecular weight compounds, 40–50 °C is suggested for extractions of essential oils by SFE [30]. As the characteristic components in *C. longa* are sesquiterpene, in this study, the extraction conditions as follows were finally applied: pressure, 200 bar; extraction temperature, 40 °C; static extraction time, 2 h, and without modifier. Three repetitions were performed to ensure the complete extraction. Finally, 320 mL of extract was yielded from 10 kg of *C. longa*. The GC-MS total ion chromatogram of extracted oil is shown in Figure 2. The contents of the investigated components in SFE extract were calculated by reference compounds, and the results were shown in Table 1. The typical volatile components account for a high content (76.0%) of SFE extract, which indicated that the current SFE condition is efficient for extraction of volatile terpenes from *C. longa*.

### 2.2. Optimization of Molecular Distillation for Purification of Standardized Typical Volatile Components

The essential oil that was obtained by SFE contained a high content of typical components of *C. longa*. However, some polar components were inevitably co-extracted, which were eluted after 15 min in the GC-MS chromatogram shown in Figure 2. Therefore, MD was applied for further purification. The distilling temperature and distilling pressure are two major factors affecting the purification of MD as the mean free path (λ_m_) was calculated by the following equation:

(1) $$\lambda_{m}=\frac{k_{B}T}{\sqrt{2}\pi d^{2}p}$$

where *k_B_* is the Boltzmann constant, *d* is molecular diameter, *p* is pressure, *T* is temperature. 

**Figure 3:**
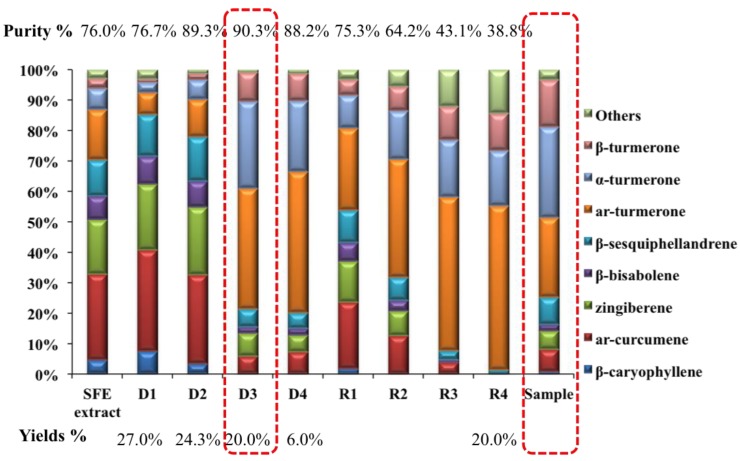
Comparison of contents, purity, and yields of distillates by molecular distillation.

The increase of distilling temperature or the decrease of distilling pressure could increase the λ_m_ of molecules [31]. In order to remove the polar components at low temperature to avoid the degree of typical volatile components, the highest vacuum that the instrument could achieve was applied and possible low temperature was optimized for the distillation. In this study, different initial temperatures for step distillation (40 °C, 50 °C, and 60 °C) were optimized for the enrichment of characteristic components from *C. longa* oil. There was no oil distilled out at 40 °C and 50 °C. Therefore, 60 °C was chosen as the initial temperature. The MD purification in the current study not only aims at obtaining the concentrated typical volatile components, but also the fractions with similar ratio of investigated components in real samples, which could be directly used for subsequent quantification. Therefore, a sequential distillation strategy was evaluated. The scheme of the distillation procedure is shown in Figure 1. Different distillates were obtained based on different temperatures. GC-MS total ion chromatograms of different distillates containing investigated components with different ratios are shown in Figure 2. The content and purity of the investigated components were calculated by reference compounds and the results are shown in Table 1. Three hundred mL of *C. longa* oil after distilling by MD yielded D1 (81 mL), D2 (73 mL), D3 (60 mL), D4 (18 mL), and R4 (60 mL), and the recovery was 97.3%. The purity of the Residues (R1–R4) is relatively low, which is mainly because they contain more polar compounds. The comparison of content, purity, and yield of distillates by MD is shown in Figure 3. D3 and D4 contained a high content of investigated components and had similar ratios of investigated components in *C. longa*. Taking both the purity and yield into the consideration, (D3—90.3% and 20.0%, D4—88.2% and 6.0%), D3 was finally chosen as STVCF.

### 2.3. Method Validation

The extracted ion chromatogram (EIC) was used for accurate quantitation of eight investigated compounds. Characteristic fragment ions, i.e., *m*/*z* 93 for β-caryophyllene, *m*/*z* 119 for ar-curcumene and zingiberene, *m*/*z* 69 for β-bisabolene and β-sesquiphellandrene, *m*/*z* 216 for ar-turmerone, *m*/*z* 111 for α-turmerone, and *m*/*z* 120 for β-turmerone, were selected for GC-MS, which are the highest abundance ions and beneficial for the separation (Table 2). The linearity, regression, and linear ranges of the investigated components were determined using the developed GC-MS method (Table 3). The results indicated a good (*R*^2^ > 0.9990) linear relationship between the amount of investigated compounds and their peak areas within the test ranges. LODs and LOQs were less than 0.85 ng and 1.92 ng, and the overall intra- and inter-day variations (RSD) of the investigated analytes were less than 1.9% and 2.2%, respectively (Table 4). For repeatability testing, the RSD of all analytes were less than 4.9%, 4.0%, and 4.9% at low, middle, and high level. The results of stability test showed the variation of analytes in solutions during the tested range is small (RSD ≤ 4.1 %), which indicated that the sample and STVCF solutions were stable under room temperature (25 °C) within 2 days. The recoveries of investigated analytes were between 98.3% and 101.1%. These data showed that the developed GC-MS method was sensitive, precise, and accurate for quantitative determination of investigated compounds in *C. longa*.

### 2.4. Quantification of Investigated Components in C. longa and Method Assessment

**Figure 4:**
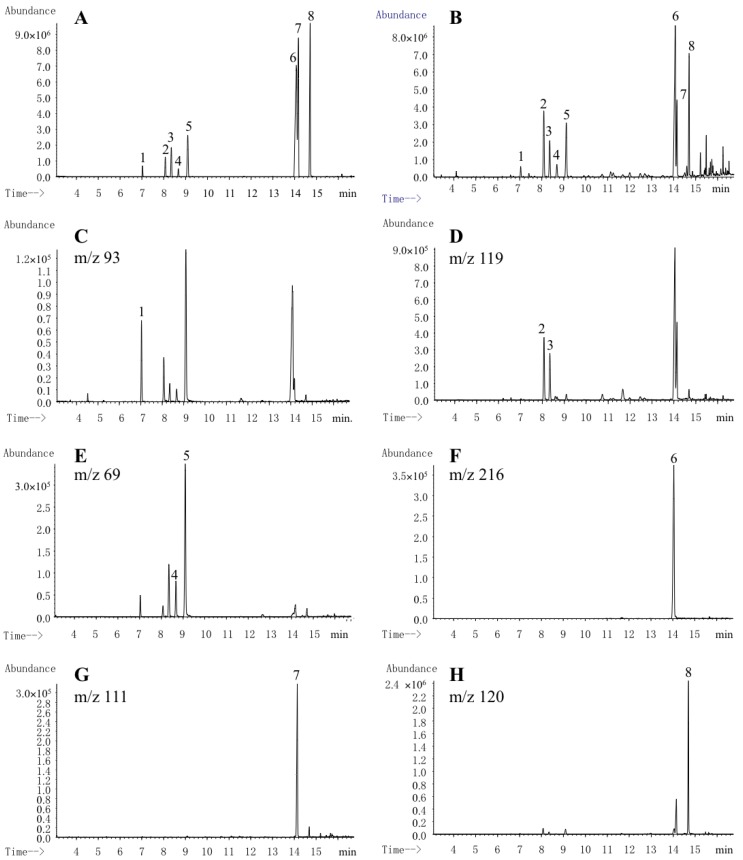
GC–MS total ion chromatogram of mixed standards (**A**), PLE extract (**B**) and the selected ion chromatograms for (**C**) β-caryophyllene, (**D**) ar-curcumene + zingiberene, (**E**) β-bisabolene + β-sesquiphellandrene, (**F**) ar-turmerone, (**G**) α-turmerone, and (**H**) β-turmerone.

The developed GC-MS method was applied for simultaneous determination of eight investigated components (β-caryophyllene, ar-curcumene, zingiberene, β-bisabolene, β-sesquiphellandrene, ar-turmerone, α-turmerone and β-turmerone) in 19 batches of *C. longa* samples. The typical total ion and extracted ion GC-MS chromatograms of mixed standards and methanol extracts of *C. longa* were shown in Figure 4. The peaks of ar-turmerone and α-turmerone could not be baseline separated in total ion chromatograms (Figure 4A,B), and the characteristic fragment ions of *m*/*z* 216 and 111 were selected for their quantitation, respectively, which could completely separate the two peaks with higher selectivity (Figure 4F,G). The identification of the investigated compounds was carried out by comparison of their retention time and mass spectra with those obtained by injecting standards (stock solutions of reference standards and STVCF) under the same conditions. Obviously, the unambiguous identification of analytes based on STVCF is easily obtained, especially when MS is not available, using the relative retention time for peak identification [32,33]. The PLE extract of 19 batches of samples were analyzed by the developed GC-MS method. The contents of eight investigated components in *C. longa* were calculated using two methods (except β-caryophyllene, which is not contained in STVCF), Method 1 and Method 2, were summarized in Table 5.

In order to evaluate the method feasibility of the STVCF-based qualification of multiple analytes, i.e., quantitation accuracy of ar-curcumene, zingiberene, β-bisabolene, β-sesquiphellandrene, ar-turmerone, α-turmerone, and β-turmerone calculated by STVCF, percent difference (PD), and *Cos* (*θ*), cosine similarity between two vectors, were employed. The calculation of PD is:

100 × (|*x*1 − *x*2|)/[(*x*1 + *x*2)/2], (2)

where *x*_1_ and *x*_2_ are the contents produced by Methods 1 and 2. The calculation of *Cos* (*θ*) is as the following equation:

(3) $$cos\left(\theta \right)\left(X,Y\right)=\frac{\sum_{i=1}^{n}X_{i}Y_{i}}{\sqrt{\sum_{i=1}^{n}{\left(X_{i}\right)}^{2}}\times \sqrt{\sum_{i=1}^{n}{\left(Y_{i}\right)}^{2}}}$$

where *X* and *Y* are the contents produced by Methods 1 and 2, and n is the number of data sets [34].

As shown in Table 5, the average PDs of eight analytes were all less than 8.5%. The *Cos* (*θ*) of ar-curcumene, zingiberene, β-bisabolene, β-sesquiphellandrene, ar-turmerone, α-turmerone, and β-turmerone were 1.000, 0.9999, 1.000, 1.000, 1.000, 0.9998, and 1.000, which demonstrated that the similarities of pairwise arrays between Methods 1 and 2 were high. Actually, the variation of analytes’ contents among different samples derived from natural material is usually high [34]. The content variation as a percentage of sample number beyond the range of 80–120% average values of β-caryophyllene, ar-curcumene, zingiberene, β-bisabolene, β-sesquiphellandrene, ar-turmerone, α-turmerone, and β-turmerone in 19 samples tested in this study were 63.2%, 63.2%, 84.2%, 73.7%, 84.2%, 42.1%, 52.6%, and 31.6%, which significantly indicated that the QC of typical volatile components in *C. longa* is crucial. Also, the data demonstrated that the variation of contents was much higher than the quantification error using STVCF for calculation. In addition, functional foods usually have been consumed for many years with good safety. The excessive pursuit of accuracy leads to an increase in time, cost, and labor required, which is impractical and unnecessary. Therefore, the quantification errors of ar-curcumene, zingiberene, β-bisabolene, β-sesquiphellandrene, ar-turmerone, α-turmerone, and β-turmerone produced by STVCF should be acceptable for QC of *C. longa*. Additionally, the pure volatile components in *C. longa* are extraordinary unstable. However, it is stable in the essential oil extracts [18]. STVCF, which has similar ratios of the investigated components in real samples, effectively simplified the analytical procedure for quantification of all the investigated components. Therefore, the established SFE and MD method for the purification of STVCF from *C. longa* and STVCF-based qualification of multiple analytes is especially suitable for the QC of volatile components in functional foods.

## 3. Materials and Methods

### 3.1. Chemicals and Materials

**Figure 5:**
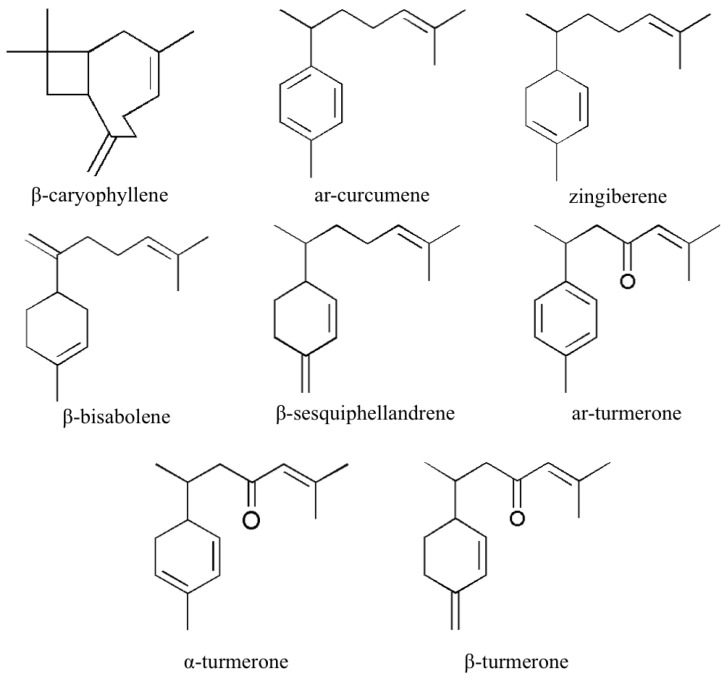
Structures of eight typical volatile compounds in *C. longa*.

β-Caryophyllene was purchased from Tokyo Chemical Industry Co., Ltd. (Tokyo, Japan). Ar-curcumene, zingiberene, β-bisabolene, β-sesquiphellandrene, ar-turmerone, α-turmerone, and β-turmerone were purified in our lab. Their structures are shown in Figure 5. The purity of all compounds is more than 95% (determined by HPLC and GC). The structures were confirmed by comparing their EI-MS and NMR data with references [35,36,37,38]. The STVCF of *C. longa*, prepared from raw materials of *C. longa* rhizome by SFE and MD, consisting of ar-curcumene, zingiberene, β-bisabolene, β-sesquiphellandrene, ar-turmerone, α-turmerone, and β-turmerone, was prepared in our lab. The content of ar-curcumene (50.3 mg/g), zingiberene (63.8 mg/g), β-bisabolene (18.4 mg/g), β-sesquiphellandrene (49.9 mg/g), ar-turmerone (339.6 mg/g), α-turmerone (245.7 mg/g), and β-turmerone (82.2 mg/g) in STVCF was calibrated with standards purified in our lab, which were prepared separately with the standards used for individual calibration curve method. The materials of *C. longa* were collected from different locations in China (Supplementary Information Table S1). The botanical origin of materials was identified by a corresponding author and the voucher specimens of these samples were deposited at the Institute of Chinese Medical Sciences, University of Macau, Macao SAR, China. Methanol was HPLC-grade from Merck (Darmstadt, Germany).

### 3.2. Supercritical Fluid Extraction of Essential Oil

Ten kilograms of *C. longa* (JH-15) was dried at 40 °C for 8 h and grounded to powder (20 mesh). One kg of the powder was packed by gauze (to avoid the contamination and clogging of pipes of SFE instrument), and then put into a SFE extraction tank (capacity, 5 L). The extraction was performed on a SFT-250 SFE/SFR system (Supercritical Fluid Technologies, Inc. Newark, DE, USA). The extraction conditions were: pressure, 200 bar; extraction temperature, 40 °C; static extraction time, 2 h. After the compressed air and CO_2_ supply was closed, and the pressure restrict valve was opened, the extract was collected using a strengthened glass bottle. Three replicates as mentioned above were performed.

### 3.3. Preparation of Standardized Typical Volatile Components Fraction by Step Molecular Distillation (MD)

A wiped-film short path distiller (POPE Scientific Inc., Saukville, WI, USA) was used for the purification of characteristic components from *C. longa* oil. Three hundred mL of essential oils extracted by SFE were distilled by a sequential distillation to obtain fractions with high purity and similar ratio of investigated components in real samples. The SFE extract were firstly put into the reception chamber (capacity, 800 mL) and different fractions were obtained by step distillation with rising temperatures (Figure 1). The first distillation was set at 60 °C; wiper rolling speed: 300 rpm; feed flow-rate: 3 mL/min; cooling temperature: 5 °C; pressure: 120 Pa. After the first distillation, two fractions were collected, called Distillate 1 (D1) and Residue 1 (R1). In the second distillation, R1 was applied for further distillation at 70 °C, and two new fractions, Distillate 2 (D2) and Residue 2 (R2), were collected. Subsequently, Distillates 3, 4 (D3, 4), and Residues 3, 4 (R3, 4) were obtained at 80 °C and 90 °C, respectively. The recovery of the obtained fractions was calculated by comparison with the total amount of loading sample. Each fraction was analyzed by the developed GC-MS method and the content of investigated components was calculated by reference compounds. The purity was also applied for estimating the enrichment capability of MD for the investigated components and was calculated by the following equation:

Purity % = 100 × total weight of investigated components in fraction/weight of fraction

### 3.4. Sample Preparation

Pressurized liquid extraction (PLE) was performed on a Dionex ASE 350 (Dionex Corp., Sunnyvale, CA, USA) system under the optimized conditions [32]. The powder of *C. longa* (0.5 g) was mixed with diatomaceous earth at the ratio of 1:1 and placed into 10 mL stainless steel extraction cell. The sample was extracted under the optimized conditions: solvent, methanol; temperature, 140 °C; particle size, 0.15–0.20 mm; static extraction time, 5 min; pressure, 1500 p.s.i.; static cycle, 1; and 60% of the flush volume. Then, the extract was transferred to a 25 mL volumetric flask (or to a 100 mL volumetric flask if the content of analytes beyond the upper limit of linearity ranges) which was made up to its volume with extraction solvent and filtered through a 0.45 μm Econo filter (Agilent Technologies, Santa Clara, CA, USA) prior to injection into the GC-MS system.

### 3.5. GC-MS Analysis

The distillates and PLE extracts were analyzed by GC-MS on an Agilent 6890 gas chromatography instrument coupled with an Agilent 5973 mass spectrometer (Agilent Technologies, Palo Alto, CA, USA). A HP-5MS capillary column (30 m × 0.25 mm, i.d.) coated with 0.25 μm film 5% phenyl methyl siloxane was used for separation. The column temperature was set at 80 °C for injection, then programmed at 20 °C/min to 150 °C held for 10 min, then at 40 °C/min to 280 °C. Split injection (2 μL) with a split ratio of 1:25 was applied. High purity helium was used as carrier gas with flow rate of 1.0 mL/min. The mass spectrometer was operated in electron-impact (EI) mode, the scan range was 35–550 amu, the ionization energy was 70 eV and the scan rate was 2.89 s per scan. The inlet and ionization source temperature were 250 °C and 280 °C, respectively.

### 3.6. Calibration Curves, Limit of Detection (LOD), and Limit of Quantification (LOQ)

Methanol stock solutions of eight reference compounds were prepared and diluted to appropriate concentrations to establish calibration curves of individual components. At least 6 concentrations of each standard were analyzed in duplicate, and then the calibration curves were constructed by plotting the peak areas versus the amount (ng) of each analyte. The quantitation of each analyte was performed based on its individual calibration curve (Method 1). Similarly, an appropriate amount of STVCF was dissolved in methanol and diluted to a series of appropriate concentrations of investigated components to establish calibration curves of individual components. At least 6 concentrations of each analyte were analyzed in duplicate, and then the calibration curves were constructed by plotting the peak areas versus the amount (ng) of each analyte. The quantitation of each analyte was performed based on its individual calibration curve (Method 2).

The stock solutions of eight reference compounds were diluted to a series of appropriate concentrations with methanol, and aliquot of the diluted solutions were injected into GC-MS for the analysis. The LOD and LOQ under the present chromatographic condition were determined at a signal-to-noise ratio (S/N) of about 3 and 10, respectively.

### 3.7. Precision, Repeatability, Stability, and Accuracy

Intra- and inter-day variations were chosen to determine the precision of the developed assay. For intra-day variability test, the mixed standards solutions were analyzed for six replicates within one day, while for inter-day variability test, the solutions were examined in duplicates for three consecutive days. Variations were expressed by RSD. 

The repeatability of the developed method was evaluated at three different levels (0.4, 0.5, and 0.6 g) of the JH-15 sample. The sample of each level was extracted and analyzed in triplicate as mentioned above. The repeatability is presented as RSD (*n* = 3).

For measurement of stability, the sample solutions (JH-15) and STVCF were stored at injection vial at room temperature. The analyses were performed after 0, 6, 12, 24, 36, and 48 h, respectively. RSD values of peak areas were calculated.

The recovery was used to evaluate the accuracy of the method. Known amounts of individual standards were added into a certain amount (0.25 g) of JH-15, and then six duplicates of the mixed samples were extracted and analyzed using the method mentioned above. The percentage recoveries were calculated by the following equation:

Recovery % = 100 × (found amount − original amount)/spiked amount

## 4. Conclusions

In this study, the SFE coupled with step MD were demonstrated to be a reliable method for preparation of STVCF from turmeric. STVCF-based qualification and quantitative evaluation of typical volatile components in turmeric was demonstrated to be credible for solving the shortage of reference volatile compounds and improving the QC of typical volatile components in turmeric and other herbal medicines. 

## Figures and Tables

**Table 1:** Contents (mg/g) of investigated components in different fractions distilled by molecular distillation.

Analyte	RT (min)	SFE Extract	D1	D2	D3	D4	R1	R2	R3	R4
β-caryophyllene	7.030	35.7	59.2	29.7	− ^a^	−	12.7	+ ^b^	−	−
ar-curcumene	8.076	221.2	263.1	264.3	53.4	63.6	171.2	85.7	18.7	+
zingiberene	8.352	139.9	171.1	201.4	67.8	49.6	103.6	54.5	+	−
β-bisabolene	8.675	61.3	71.9	78.2	19.5	19.7	48.3	24.5	4.0	−
β-sesquiphellandrene	9.104	93.8	108.3	130.9	53.0	46.6	83.5	51.3	14.3	6.2
ar-turmerone	14.080	128.2	57.4	111.3	360.9	413.3	209.1	263.4	248.3	244.2
α-turmerone	14.180	54.8	26.7	57.6	261.1	208.0	84.4	108.4	92.6	82.3
β-turmerone	14.715	25.2	9.1	19.9	87.4	80.9	40.5	53.9	53.4	55.1
Purity		76.0%	76.7%	89.3%	90.3%	88.2%	75.3%	64.2%	43.1%	38.8%

^a^ Undetected. ^b^ Under the limit of quantitation.

**Table 2:** Mass data of eight characteristic typical volatile compounds in *C. longa*.

Compound	EIC	Mass Data
β-caryophyllene	93	204(M+, 12), 161(38), 133(95), 119(35), 105(54), 93(100), 91(88), 79(81), 69(79), 55(36), 41(77)
ar-curcumene	119	202(M+, 31), 145(25), 132(98), 131(26), 120(27), 119(100), 117(23), 105(47), 91(24), 41(22)
zingiberene	119	204(M+, 11), 119(100), 93(82), 91(35), 77(23), 69(26), 56(10), 55(9), 41(19)
β-bisabolene	69	204(M+, 29), 161(23), 135(13), 121(7), 119(69), 109(28), 93(81), 79(37), 69(100), 67(39), 41(71)
β-sesquiphellandrene	69	204(M+, 27), 161(49), 133(36), 120(36), 93(64), 91(55), 77(37), 69(100)
ar-turmerone	216	216(M+, 30), 201(20), 132(20), 120(7), 119(72), 117(14), 115(8), 105(11), 91(14), 83(100), 55(15)
α-turmerone	111	218(M+, 4), 120(55), 119(50), 111(27), 105(97), 93(19), 91(32), 85(15), 83(100), 77(23), 55(23)
β-turmerone	120	218(M+, 2), 121(10), 120(100), 105(15), 93(3), 92(6), 91(13), 83(25), 79(4), 77(7), 55(9)

**Table 3:** Regression data, LOD, and LOQ of the investigated compounds.

Analytes	Linear Regression Data			LOD (ng)	LOQ (ng)
	Regressive Equation	Test Range (ng)	*R* ^2^		
β-caryophyllene	y = 9890.2 x + 2434.0	0.77–49.06	0.9993	0.21	0.37
ar-curcumene	y = 23935.5 x + 466.0	0.59–28.52	0.9992	0.13	0.28
zingiberene	y = 17561.3 x − 17360.2	2.21–106.01	0.9991	0.82	1.87
β-bisabolene	y = 14340.6 x − 5571.0	0.71–22.76	0.9995	0.16	0.30
β-sesquiphellandrene	y = 10306.9 x − 9855.2	1.20–28.83	0.9991	0.27	0.58
ar-turmerone	y = 8418.0 x − 663.8	1.45–46.49	0.9992	0.32	0.89
α-turmerone	y = 4553.1 x − 212.3	8.89–106.68	0.9994	0.85	1.92
β-turmerone	y = 38206.5 x − 95098.8	1.26–120.68	0.9990	0.28	0.53

**Table 4:** Result of accuracy, precision, repeatability and stability.

Analyte	Recovery (%, RSD, *n* = 6)	Precision (RSD, %, *n* = 6)		Repeatability (RSD, %, *n* = 3)		Stability (RSD, %, *n* = 6)				
		Intra-day	Inter-day	Low	Middle	High	Sample Solution		STVCF Solution	
							24 h	48 h	24 h	48 h
β-caryophyllene	98.3 (2.3)	1.2	1.5	4.6	1.9	3.4	1.2	1.8	− ^a^	−
ar-curcumene	100.5 (2.8)	1.4	1.4	4.4	1.5	2.7	1.6	2.1	2.8	2.9
zingiberene	100.9 (2.8)	1.6	1.7	4.1	3.0	2.8	0.5	2.5	1.0	2.1
β-bisabolene	99.9 (3.2)	0.9	1.5	3.1	1.9	4.4	0.3	3.0	2.5	2.7
β-sesquiphellandrene	100.7 (3.3)	1.1	1.3	4.4	2.1	2.5	0.7	2.0	1.5	2.6
ar-turmerone	99.9 (4.0)	1.8	2.1	4.9	3.8	3.8	1.2	2.6	1.4	3.4
α-turmerone	100.3 (4.2)	1.9	1.8	4.8	3.3	4.9	0.8	1.2	1.7	4.1
β-turmerone	101.1 (4.7)	1.7	2.2	4.7	4.0	4.4	0.9	3.0	0.2	3.7

^a^ Not applicable.

**Table 5:** Comparison for the contents (mg/g) of investigated components in *C. longa* calculated by individual reference compounds (M1) and STVCF calibration curves (M2).

Samples	β-caryophyllene			ar-curcumene			zingiberene			β-bisabolene			β-sesquiphelland-rene			ar-turmerone			α-turmerone			β-turmerone			Total
	M1	M2	PD (%)	M1	M2	PD (%)	M1	M2	PD (%)	M1	M2	PD (%)	M1	M2	PD (%)	M1	M2	PD (%)	M1	M2	PD (%)	M1	M2	PD (%)	
JH-1	+ ^a^	− ^b^	-	0.9	1.0	4.7	2.9	2.8	4.2	0.4	0.4	1.4	2.4	2.2	6.1	11.3	11.6	2.3	27.8	28.8	3.4	10.0	9.4	6.2	55.7
JH-2	+	−	-	0.7	0.8	5.0	2.7	2.6	4.9	0.3	0.3	2.5	2.0	1.9	7.2	9.5	9.7	2.1	23.6	24.1	2.1	8.4	7.9	6.6	47.2
JH-3	0.3	−	-	3.1	3.3	3.9	2.7	2.5	5.1	1.0	0.9	1.6	3.8	3.7	4.0	11.4	11.6	2.3	13.0	12.3	5.6	6.7	6.2	7.2	41.9
JH-4	+	−	-	0.7	0.7	5.1	2.1	2.0	7.9	0.3	0.3	3.2	1.8	1.7	7.8	9.8	10.0	2.2	22.2	22.6	1.5	8.3	7.7	6.6	45.2
JH-5	1.2	−	-	2.4	2.5	4.0	11.8	12.2	3.3	1.3	1.3	2.1	6.8	6.7	2.5	8.1	8.3	2.0	31.1	32.5	4.3	9.1	8.5	6.4	71.8
JH-6	0.8	−	-	2.3	2.3	4.1	11.7	12.1	3.3	1.4	1.3	2.2	6.7	6.5	2.5	7.3	7.4	1.9	29.2	30.3	3.8	8.4	7.9	6.6	67.6
JH-7	0.8	−	-	1.2	1.3	4.4	6.4	6.5	1.3	0.7	0.7	0.8	3.5	3.3	4.3	4.4	4.5	1.2	17.6	17.4	1.1	4.7	4.3	8.5	39.4
JH-8	1.6	−	-	1.7	1.8	4.2	15.0	15.6	3.8	1.5	1.4	2.3	7.5	7.4	2.3	7.2	7.3	1.9	37.9	40.0	5.4	9.9	9.3	6.2	82.2
JH-9	1.5	−	-	2.3	2.3	4.1	14.7	15.3	3.7	1.5	1.5	2.4	7.7	7.6	2.3	8.8	9.0	2.1	36.4	38.4	5.2	10.2	9.6	6.1	83.2
JH-10	1.4	−	-	1.9	2.0	4.1	13.3	13.8	3.5	1.4	1.4	2.2	7.2	7.0	2.4	8.4	8.6	2.0	38.3	40.5	5.5	10.8	10.2	6.0	82.8
JH-11	1.2	−	-	1.9	1.9	4.2	12.6	13.0	3.4	1.3	1.3	2.1	6.5	6.4	2.6	7.4	7.5	1.9	31.1	32.5	4.3	8.7	8.1	6.5	70.7
JH-12	1.4	−	-	1.8	1.8	4.2	11.9	12.3	3.3	1.2	1.2	2.0	6.5	6.3	2.6	6.7	6.8	1.8	33.2	34.8	4.7	8.8	8.2	6.5	71.5
JH-13	1.1	−	-	2.1	2.1	4.1	11.9	12.3	3.3	1.3	1.3	2.1	6.7	6.5	2.5	7.3	7.4	1.9	31.5	32.9	4.3	9.0	8.4	6.4	70.8
JH-14	1.2	−	-	1.7	1.7	4.2	13.4	13.9	3.6	1.3	1.3	2.1	6.7	6.5	2.5	6.5	6.6	1.8	31.9	33.4	4.4	8.6	8.0	6.5	71.3
JH-15	0.5	−	-	4.2	4.4	3.8	3.0	2.9	3.7	0.9	0.9	1.4	4.3	4.1	3.6	16.9	17.3	2.5	6.1	4.6	8.1	6.5	6.0	7.3	42.4
JH-16	0.9	−	-	1.9	2.0	4.1	10.4	10.7	3.0	1.1	1.1	1.9	5.6	5.5	2.9	6.9	7.1	1.8	28.1	29.1	3.5	8.0	7.5	6.7	63.0
JH-17	0.8	−	-	2.7	2.8	4.0	8.9	9.1	2.5	1.2	1.1	1.9	5.9	5.8	2.8	11.8	12.1	2.3	21.3	21.6	1.1	8.4	7.8	6.6	61.0
JH-18	0.6	−	-	1.0	1.0	4.7	7.3	7.4	1.8	0.7	0.7	0.7	3.6	3.4	4.2	9.8	10.0	2.2	18.9	18.8	0.2	6.6	6.2	7.2	48.5
JH-19	0.5	−	-	0.8	0.8	4.9	6.4	6.5	1.3	0.6	0.6	0.3	3.0	2.9	4.9	8.1	8.3	2.0	16.8	16.6	1.6	5.9	5.5	7.6	42.2
Cos (θ)	−			1.000			0.9999			1.000			1.000			1.000			0.9998			1.000			

^a^ Under the limit of quantitation; ^b^ Not applicable.

## Supplementary Materials

The following are available online, Table S1: Summary for the tested samples of *C. longa*.
