# Supplementary material for: Preparation and Application of Standardized Typical Volatile Components Fraction from Turmeric (Curcuma longa L.) by Supercritical Fluid Extraction and Step Molecular Distillation
Source: Molecules. 2018 Jul 23;23(7):1831. doi: 10.3390/molecules23071831 (PMC6099931; doi:10.3390/molecules23071831)
Supplement: Supplementary file 1 [file molecules-23-01831-s001.pdf]

Table S1 Summary for the tested samples of *C. longa*

No.	Code	Samples	Sources	Collection date
1	JH-1	*C. longa*	Wenshan, Yunana	2008
2	JH-2	*C. longa*	Chengdu, Sichuan	2008
3	JH-3	*C. longa*	Chongzhou, Sichuan	2007
4	JH-4	*C. longa*	Chengdu, Sichuan	2008
5	JH-5	*C. longa*	Chengdu, Sichuan	2014
6	JH-6	*C. longa*	Chengdu, Sichuan	2014
7	JH-7	*C. longa*	Chengdu, Sichuan	2014
8	JH-8	*C. longa*	Chengdu, Sichuan	2014
9	JH-9	*C. longa*	Chengdu, Sichuan	2014
10	JH-10	*C. longa*	Chengdu, Sichuan	2014
11	JH-11	*C. longa*	Chengdu, Sichuan	2014
12	JH-12	*C. longa*	Chengdu, Sichuan	2014
13	JH-13	*C. longa*	Chengdu, Sichuan	2014
14	JH-14	*C. longa*	Chengdu, Sichuan	2014
15	JH-15	*C. longa*	Chengdu, Sichuan	2009
16	JH-16	*C. longa*	Chengdu, Sichuan	2014
17	JH-17	*C. longa*	Chengdu, Sichuan	2008
18	JH-18	*C. longa*	Jiaqing, Sichuan	2008
19	JH-19	*C. longa*	Xinyi, Sichuan	2008
